# Effects of Graphene Oxide on Phosphorus Uptake in the Arbuscular Mycorrhizal Symbiosis of *Medicago sativa* L

**DOI:** 10.3390/plants15071088

**Published:** 2026-04-01

**Authors:** Shulan Zhao, Hongda Wei, Lian Duo

**Affiliations:** Tianjin Key Laboratory of Animal and Plant Resistance, College of Life Sciences, Tianjin Normal University, Tianjin 300387, China; zhaosl_tjnu@126.com (S.Z.);

**Keywords:** graphene oxide, *Medicago sativa* L., arbuscular mycorrhizal symbiosis, phosphorus transport

## Abstract

The majority of terrestrial plant species establish below-ground interconnections via arbuscular mycorrhizal (AM) mycelium, thereby forming extensive common mycorrhizal networks (CMNs). CMNs serve as critical infrastructure for nutrient acquisition, mediating soil nutrient capture and distribution. In nitrogen-fixing plants, phosphorus (P) transport is particularly dependent on functional CMNs. The rapid expansion in graphene oxide (GO) production and its broad application have raised significant ecological concerns, particularly regarding its potential impacts on terrestrial ecosystems. Despite these concerns, the impact of GO on P transport dynamics within legume–arbuscular mycorrhizal fungi (AMF) symbioses remains critically scarce. This study established a symbiotic system using the model nitrogen-fixing legume *Medicago sativa* L. and AMF. This experimental system enabled a comprehensive assessment of GO effects on rhizosphere P mobilization, plant P acquisition, CMNs architecture, fungal community composition, and expression of key P transporter genes. Our results demonstrated that high GO concentrations significantly altered rhizosphere properties, increasing pH while reducing organic acid content and alkaline phosphatase activity. Furthermore, GO exposure significantly inhibited root growth, mycorrhizal colonization rates, and plant P acquisition efficiency. Additionally, GO exposure altered AMF community composition, reduced rhizosphere microbial diversity, and suppressed P metabolism gene expression. Specifically, 0.6% GO induced significant downregulation of *MsCS* and *GigmPT* by 83.5% and 62.3%, respectively. This indicates that GO impairs plant P uptake by disrupting the core pathway involving *GigmPT* and *MsCS*, triggering P stress in *M. sativa*. Collectively, these findings provide compelling evidence that GO exposure disrupts legume–AMF symbiotic integrity, ultimately impairing P transport efficiency.

## 1. Introduction

GO nanomaterials have emerged as one of the most important nanomaterials discovered in the 21st century. As a derivative of graphene, GO consists of graphene sheets functionalized with numerous oxygen-containing groups on both their basal planes and edges [1]. Beyond its remarkable mechanical and optical properties, GO exhibits distinctive characteristics that differentiate it from graphene. Its exceptional versatility has driven widespread applications in various fields, including electronics, energy storage, composite materials, biomedicine, and environmental protection [2]. However, as the increase in GO production and utilization, environmental concerns regarding potential risks associated with its release have also been intensified [3,4].

Extensive studies have established that graphene-based nanomaterials can exert detrimental effects on plants, microorganisms, animals and human cells [5,6]. Nanomaterial toxicity is primarily driven by reactive oxygen species (ROS) generation, which induces oxidative damage to diverse biomolecules, including lipids, proteins, and nucleic acids [7]. For instance, GO suppresses algal growth through shading effect, membrane damage via oxidative stress and penetration, and nutrient depletion from the culture medium [8]. GO triggers histological alterations in zebrafish livers and modulates gene expression by inhibiting immune-associated signaling pathways [9]. GO-based nanocomposites act as promising antibacterial agents for agriculture and biomedical applications [10,11]. Although the ecotoxicological risks of GO are well-documented, current research has focused predominantly on individual model organisms.

Arbuscular mycorrhizal fungi (AMF) are widely distributed across all soil types and establish mutualistic symbioses with roughly 80% of terrestrial plant species (over 250,000 total) in natural ecosystems [12,13]. Extraradical AMF mycelium penetrates soil micropores inaccessible to plant roots, significantly expanding root absorptive surface area and enhancing host plant acquisition of mineral nutrients [14]. Nearly all nitrogen (N)-fixing plants form symbioses with AMF. Legumes, one of the most widespread plant families, form root nodules that host N-fixing rhizobia, enabling biological N fixation. Symbiotic nitrogen fixation (SNF) dominates about 65% of total biological N fixation and serves as a critical nitrogen source in agricultural systems [15,16]. Over long-term evolution, AMF has developed tight mutualistic symbioses with legumes [17], giving rise to CMNs that mediate water and nutrient allocation as well as inter-organismal chemical signaling [18].

Phosphorus (P) is an essential macronutrient element for plant growth and metabolism, and a core constituent of key biomolecules including nucleic acids, phospholipids, and adenosine triphosphate (ATP). P mediates diverse essential physiological processes, including energy metabolism, cell division, enzymatic catalysis, genetic information transfer, photosynthesis, and stomatal signaling [19]. Plants primarily take up soil P in the form of inorganic phosphate anions (Pi, H_2_PO_4_^−^/HPO_4_^2−^) via membrane transporters [20]; however, most soil P occurs in insoluble forms, including Fe-P, Ca-P, Al-P, and organic P complexes, resulting in P limitation for plants [19,21]. P deficiency represents a primary constraint on SNF and legume crop productivity [22,23]. To adapt to low P availability, plants have evolved diverse morphological and physiological strategies, including exudation of organic acids and phosphatases into the rhizosphere, maintenance of nodule P homeostasis, and induction of high-affinity phosphate transporters [23,24]. Organic acids exuded from plant roots acidify rhizosphere, promoting the dissolution of insoluble P compounds, and thereby enhancing soil P bioavailability. Organic acids exhibit strong affinity for divalent and trivalent cations, chelating Al^3+^, Fe^3+^ and Ca^2+^ to liberate phosphate ions for plant uptake. Under P deficiency, *M. sativa* secretes more citric acid, which is synthesized from oxaloacetate (OAA) and acetyl CoA and catalyzed by citrate synthase (*CS*) [25]. Overexpression of the *MsCS* gene enhances P acquisition in *M. sativa* [26]. AMF inoculation colonizes host plant roots and forms extensive CMNs, enhancing P use efficiency even under P deficiency [27]. AMF upregulates *MsCS* expression and induces high-affinity phosphate transporters in the host, enhancing organic acid exudation and thereby improving P uptake and tolerance to P deficiency [28,29]. This study proposes the hypothesis that GO initially impairs legume P acquisition by disrupting mycorrhizal formation and functions—specifically through suppressing *GigmPT* and *MsCS* expression, thereby inducing P stress.

Growing attention has recently been directed toward understanding interactions between graphene nanomaterials and plants. Such studies have primarily characterized plant responses to graphene-based materials at the individual organism level, focusing on growth, physiological, biochemical, and molecular parameters [30]. Therefore, the impacts of GO on P transport in AMF symbiosis remain poorly understood and warrant detailed mechanistic investigation. *M. sativa* is a dominant forage crop in temperate grassland systems, distinguished by its broad adaptability, high productivity, superior palatability, and exceptional nutritional value. This study aimed to investigate the impacts of GO on P transport in mycorrhizal symbiosis by using *M. sativa* as a model species. Our findings provide mechanistic insights into GO toxicity toward CMNs.

## 2. Results

### 2.1. Effects of GO on Soil Environment of AMF Symbiosis

**Figure 1:**
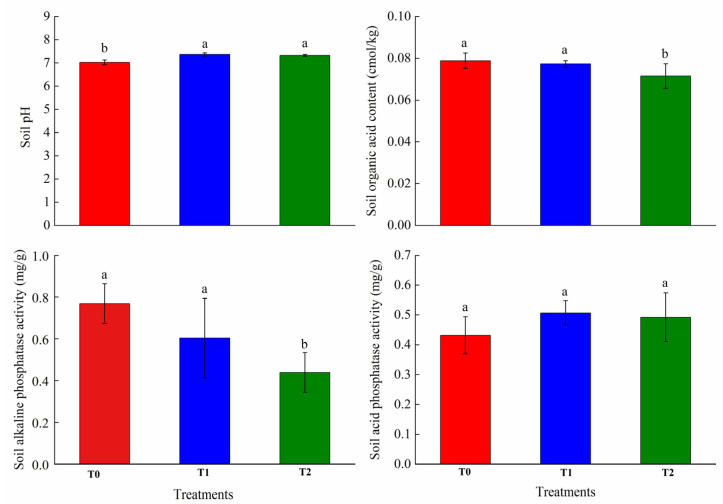
GO impacts on soil pH, organic acid content, and phosphatase activity. Each bar represents the mean ± SD (*n* = 4). Different lowercase letters above the error bars represent significant differences among treatments (*p* < 0.05) by the Duncan’s multiple range test. T0: Control; T1: 0.3% GO; T2: 0.6% GO.

GO treatment significantly altered rhizosphere soil properties, as shown in Figure 1, by increasing pH while reducing organic acid content (9.3% decrease) and alkaline phosphatase activity (42.8% decrease) at the T2 treatment. Notably, these effects displayed dose-dependence, with the T1 treatment showing no significant differences relative to the untreated control (*p* > 0.05). Soil acid phosphatase activity remained unchanged across all treatments.

### 2.2. AMF Symbiosis and Phosphorus Acquisition

**Figure 2:**
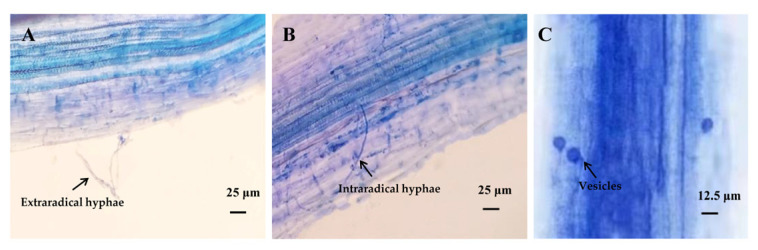
AMF colonization in *M. sativa* roots. (**A**) extraradical hyphae, (**B**) intraradical hyphae, (**C**) vesicles.

**Figure 3:**
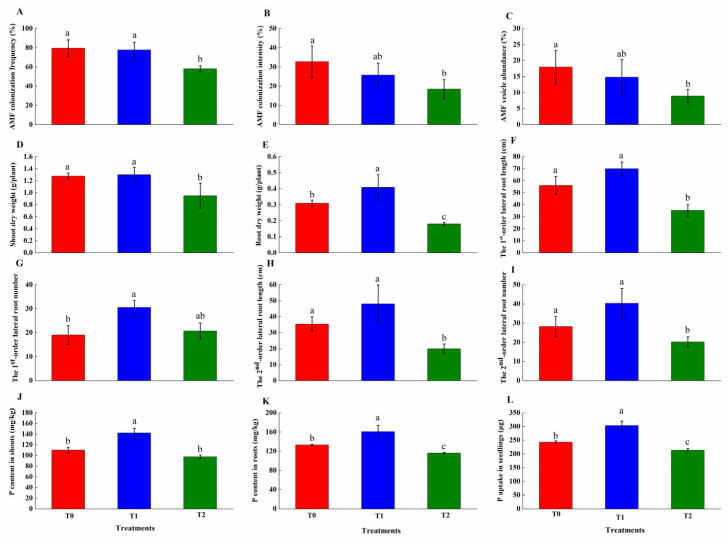
Effects of GO on AMF colonization, host biomass, root growth, and P acquisition. (**A**) AMF colonization frequency. (**B**) AMF colonization intensity. (**C**) AMF vesicle abundance. (**D**) Shoot dry weight. (**E**) Root dry weight. (**F**) The 1st-order lateral root length. (**G**) The 1st-order lateral root number. (**H**) The 2nd-order lateral root length. (**I**) The 2nd-order lateral root number. (**J**) P content in shoots. (**K**) P content in roots. (**L**) P uptake in seedlings. Each bar represents the mean ± SD (*n* = 4). Different lowercase letters above the error bars represent significant differences among treatments (*p* < 0.05) by the Duncan’s multiple range test. T0: Control; T1: 0.3% GO; T2: 0.6% GO.

Microscopic observation confirmed successful AMF colonization in *M. sativa* roots under all treatments, with typical symbiotic structures observed, including extraradical hyphae, intraradical hyphae, and vesicles (Figure 2). However, GO exposure suppressed mycorrhizal colonization parameters in a dose-dependent manner (Figure 3). The T2 treatment significantly reduced colonization frequency, intensity, and vesicle abundance by 21.32%, 14.28%, and 9.06% (*p* < 0.05), respectively. Notably, T1 did not differ significantly from the control in any colonization metrics (*p* > 0.05).

GO exposure exerted dose-dependent effects on *M. sativa* growth parameters (Figure 3). The T2 treatment significantly inhibited shoot and root biomass accumulation, reducing shoot dry weight by 25.7% and root dry weight by 42.1%, relative to controls. In contrast, T1 enhanced root growth, increasing root dry weight by 32%. Shoot biomass under T1 treatment showed no significant difference from the control. Root architecture analysis revealed significant alterations in lateral root development following GO exposure (Figure 3). T2 reduced the length of 1st and 2nd-order lateral roots and 2nd-order lateral root number by 37.1%, 43.4%, and 28.4%, respectively. In contrast, T1 increased 1st-order lateral root number by 60.5%. Figure 3 demonstrates that the T1 treatment significantly enhanced P acquisition, increasing shoot P concentration by 30%, root P concentration by 20.8%, and total P by 25% relative to controls. However, T2 significantly reduced root P concentration and total P by 12.7% and 12.1%, respectively.

### 2.3. Diversity and Composition of AMF Communities in Rhizosphere Soil

AMF diversity in rhizosphere differed significantly across all treatments (Table 1). Species richness (Sobs index), estimated richness (Chao1 index), and community diversity (Shannon index) all reached their highest values in soil amended with T1. T2 also increased AMF species richness relative to controls, as quantified by the Sobs index. No significant differences were observed in the Simpson index across all treatments.

**Figure 4:**
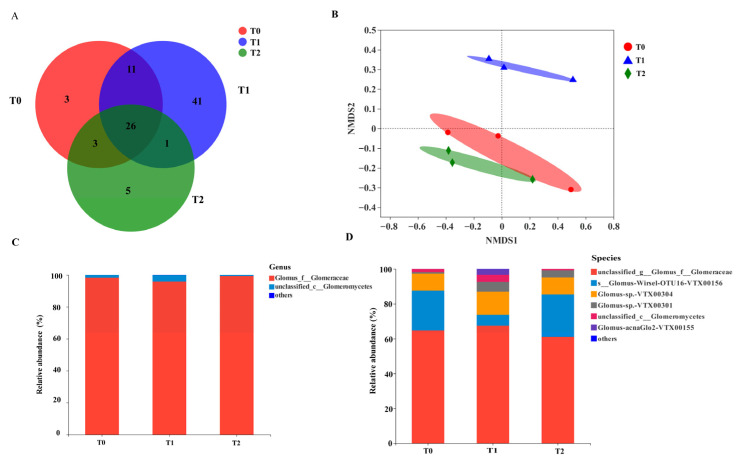
The composition and diversity of AMF communities in rhizosphere soil. (**A**) Venn diagram. (**B**) NMDS analysis. (**C**) Composition of AMF communities at genus level. (**D**) Composition of AMF communities at species level. T0: Control; T1: 0.3% GO; T2: 0.6% GO.

High-throughput sequencing yielded 157 operational taxonomic units (OTUs) across all treatments (Figure 4). Of these, 43 OTUs were detected in T0 (control), 79 in T1, and 35 in T2. There were 26 OTUs shared among all the treatments (16.5% of total). The T1 treatment exhibited the highest number of unique OTUs (41; 26.1% of total) and showed the strongest community overlap with T0 (11 shared OTUs). In contrast, only one OUT was shared between T1 and T2. NMDS ordination (stress = 0.056) revealed clear compositional differences between the high-diversity T1 community and both T0 and T2 assemblages (Figure 4).

Community composition analysis revealed overwhelming dominance of the genus *Glomus*, with relative abundance exceeding 95% across all treatments (Figure 4). The highest abundance of *Glomus* was found in the T2 treatment. At the species level, the AMF community was predominated by unclassified_*g_Glomus_f_Glomeraceae*, *Glomus* sp. (VTX00156) and *Glomus* sp. (VTX00304). Within the tested GO concentration range, the abundance of unclassified_*g_Glomus_f_Glomeraceae*, *Glomus* sp. (VTX00304), *Glomus* sp. (VTX00301), and unclassified_*c_Glomeromycetes* showed initially increasing and then decreasing activity with increasing GO concentrations. Notably, *Glomus* sp. (VTX00155) was nearly absent (<0.1% abundance) in both T0 and T2 treatments (Figure 4).

### 2.4. Gene Expression of MsCS and GigmPT

**Figure 5:**
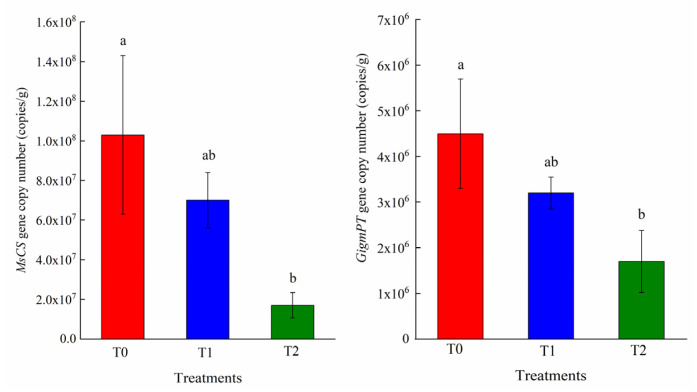
The expression of citrate synthase gene *MsCS* and phosphate transporter gene *GigmPT*. Each bar represents the mean ± SD (*n* = 3). Different lowercase letters above the error bars represent significant differences among treatments (*p* < 0.05) by the Duncan’s multiple range test. T0: Control; T1: 0.3% GO; T2: 0.6% GO.

The response of AMF-colonized seedlings to GO exposure was strongly associated with altered expression of P transport-related genes (Figure 5). Quantitative analysis revealed significant GO-induced downregulation of *MsCS* and *GigmPT* expression at T2, with reductions of 83.5% and 62.3%, respectively, relative to controls. Although both *MsCS* and *GigmPT* expression in T1 showed decreasing trends relative to controls, these changes were not statistically significant.

## 3. Discussion

The roots and hyphae of mycorrhizal plants secrete low-molecular-weight organic acids (LMWOAs) and protons into the rhizosphere, driving the dissolution of sparingly soluble P in the soil. This process increases soil P availability and enhances plant P acquisition [31]. Carboxylate exudation, including citrate, mediated by both AM symbiosis and root hairs, shapes the composition of the bacterial microbiome. AMF and their associated microbiota played a key role in releasing significant quantities of LMWOAs, which mobilize P from sparingly available soil pools [31]. In addition, phosphatase activities contributed to enhanced soil P cycling and improved plant P nutrition [32]. AMF inoculation significantly enhances phosphatase activity secreted by host plant roots [33]. AMF exudates involved in P mobilization include phosphatases, protons, phenolic compounds, and organic ligands [34]. In the present study, T2 significantly increased soil pH, while decreasing organic acid content and alkaline phosphatase activity, thereby reducing the plant P uptake. However, T1 treatment did not affect organic acid content and alkaline phosphatase activity. The biphasic response of GO—stimulatory at 0.3% but inhibitory at 0.6%—reflects its dual role in modulating soil chemistry and microbial activity. Low concentrations may enhance nutrient availability and fungal symbiosis, while high concentrations likely disrupt soil physical integrity and root exudate composition, thereby impairing P transport efficiency [35]. One important reason may be that high doses of GO cause serious damage to host plant roots and AMF. GO can induce the formation of ROS, which damages the cellular components—including the nucleus, membrane, and mitochondria—and may ultimately lead to cell death [36]. Furthermore, the lamellar structure of GO produces sharp edges that can inflict irreversible physical damage on microorganisms. At high concentrations, increased frequency of GO-AMF interactions intensifies these detrimental effects on fungal cells and hyphae [37].

AMF represent key beneficial soil microorganisms that establish mutualistic symbioses with the roots of more than two-thirds of terrestrial plant species [34]. AMF are widespread among N-fixing plants, forming dual mutualistic associations—including both legume–rhizobium and legume–AM symbioses [17]. In the present study, a functional symbiotic association was successfully established between AMF and the host plant. Colonization frequency ranged from 58.3% to 79.6%, mycorrhizal colonization intensity varied between 18.5% and 32.7%, and vesicle abundance ranged from 9.0% to 18.0%. However, high concentration of GO significantly inhibited AMF colonization, demonstrating that GO exposure impairs the symbiotic affinity and ecological compatibility between AMF and their host plants. Similarly, silver nanoparticles (AgNPs) significantly reduced mycorrhizal colonization, vesicle abundance, and arbuscule formation, with smaller particle sizes exerting inhibitory effects than larger ones [38]. Exposure to 0.5% GO also induced significant reductions in AMF colonization [39]. The adverse effects of nanoparticles are primarily attributable to their antifungal activities, including adhesion to fungal cell surfaces, physical damage to cell walls and membranes, excessive ROS accumulation, and consequent inhibition of host plant growth [40].

P is an essential macronutrient critically required for plant growth, morphological development, and normal physiological metabolic processes [41]. It participates in diverse cellular functions, including energy transduction, protein regulation, photosynthesis, and respiration, and serves as a core component of vital biological macromolecules, such as nucleic acid, ATP, and phospholipid [42]. Although P is widely abundant in agricultural soils, the vast majority exists in biologically unavailable forms—insoluble inorganic phosphorus (Pi) and organic phosphorus (Po). This condition renders P deficiency a major limiting factor for plant growth and sustainable agricultural production [43]. As symbionts to approximately 80% of terrestrial plants, AMF significantly enhance plant root P uptake and promote plant growth and development by improving root morphological traits—including root length, average diameter, surface area, volume, and branching [33]. The improvement and optimization of root architectural traits can enhance the acquisition efficiency of rhizospheric water and bioavailable nutrients [34]. AMF inoculation has been shown to improve root morphology and increase root hair length in tea seedling. In contrast, prior studies have demonstrated that GO exposure disrupts root morphology, inhibits root elongation, and reduces root biomass [41,42]. Root architecture analysis further revealed that taproot length remained unaffected, whereas lateral root development was significantly suppressed. This specific impairment of the fine root architecture—critical for nutrient foraging—is a documented response to GO stress. The results of this study demonstrate that GO treatment significantly reduced host plant biomass, concurrently impairing root morphology and architecture. These effects inhibited P acquisition through mycorrhizal symbiosis and led to a marked reduction in host tissue P content—most pronounced within the root system [44]. Consistent with these findings, GO was observed to accumulate in root vacuoles, resulting in significant suppression of taproot elongation and lateral root proliferation in wheat seedlings [45].

In symbiotic leguminous plants, the exudation of large quantities of organic acid anions by roots under P deficiency represents an efficient adaptive strategy for mobilizing insoluble P sources [34]. Citrate synthase (*CS*) catalyzes the condensation of oxaloacetate (OAA) and acetyl CoA to produce citrate and is a key enzyme in citric acid synthesis [26]. Previous research has demonstrated that overexpression of *CS* genes can significantly enhance citrate exudation and subsequently improve P acquisition in plants [46]. For example, overexpression of *CS* in *Brassica napus* significantly increased both the biosynthesis and exudation of citrate [26]. In the symbioses between AMF and their host plants, P absorption from soil by AMF represents the first step in transferring P to the host. AMF inherently encodes phosphate transporter genes that mediate the uptake of inorganic phosphate from soil into the extraradical mycelium [47]. *GigmPT* functions as a high-affinity phosphate transporter essential for AM symbiosis and is closely involved in activating the phosphate signaling pathway [47]. This transporter is constitutively expressed in both extraradical and intraradical mycelial networks, mediating phosphate uptake at the fungus–soil interface, and thereby influencing fungal growth and arbuscular development within host plant roots. In this study, seedlings exposed to T2 showed lower transcriptional expression level of *MsCS* and *GigmPT* in root tissues compared to controls—findings consistent with the observed decreases in root-derived organic acid secretion and plant P acquisition efficiency. Similarly, exposure to GO has been demonstrated to elicit the transcriptional downregulation of genes functionally associated with root morphogenesis and developmental processes in *Arabidopsis thaliana* [48]. GO also modulates the expression of phosphate transporter genes, thereby influencing the uptake, translocation, and biotransformation of arsenic (As) in plants [49]. Collectively, these findings demonstrate that high-concentration of GO exhibits genotoxic effects in plants and can modulate the transcriptional expression of functionally relevant genes involved in nutrient acquisition, stress response, and developmental processes.

The AMF community in the rhizosphere was predominantly dominated by the genus *Glomus*, indicating that the vast majority of AM fungal taxa forming symbiosis with *M. sativa* belong to the family *Glomeraceae*, specifically the genus *Glomus*. This genus exhibits the capacity to reproduce asexually via mycelial fragments and root-derived propagules, possesses high sporulation potential, and can establish stable symbiotic relationships with *M. sativa*—traits that collectively enhance its adaptability to adverse environmental conditions [50]. Previous studies have demonstrated that nanomaterials can exert adverse effects on soil microbial communities, with such effects manifesting as a significant, concentration-dependent reduction in microbial diversity [51]. For example, chronic exposure of soil to 2.0 mg/g ZnO NPs over a 30-day incubation period significantly decreased soil microbial community diversity and altered soil bacterial community composition [52]. The shifts observed in the fungal community are most likely attributable to nanomaterial-triggered cellular stress in AMF, which operates mainly via two distinct pathways: physical impairment of cellular membranes and induction of oxidative stress [7,37]. Within the context of this study, the high concentration of 0.6% GO provoked adverse effects on AMF inhabiting the rhizosphere soil of the host species, ultimately contributing to diminished richness and diversity within the fungal community, as well as substantial modifications to its structural composition [53]. Additionally, GO exposure triggered discernible changes in soil properties, particularly a significant rise in rhizosphere pH. This pH shift constitutes an important environmental determinant shaping AMF spore development, species colonization dynamics, and community assembly processes, which in turn underpins the observed modifications to AMF community structure. Notably, no *Glomus* species are reported to inhibit plant P uptake; our findings—including altered abundance of *Glomus* sp. VTX00156 and VTX00304, and the near-absence of VTX00155 under high GO—broaden our understanding of *Glomus* functional diversity, laying the foundation for future in-depth investigations. These observed structural and functional changes imply that GO exposure at this tested concentration surpasses the stress tolerance threshold of most indigenous AMF symbionts, thereby posing potential risks of disrupting critical mycorrhizal-mediated ecosystem services in terrestrial habitats [54].

## 4. Materials and Methods

### 4.1. Materials

GO was obtained from Tanfeng Graphene Tech Co., Ltd. (Suzhou, China), with a nominal purity of 96%. As comprehensively characterized in our previous paper [55], GO exhibits a well-organized crystalline structure with the presence of oxygen-containing groups.

CMNs are ubiquitous in natural vegetation systems, with the realization of key ecological functions (i.e., material cycling and energy flow) inherently dependent on these below-ground connectivity networks [56]. However, establishment of pot-based experimental systems disrupts natural CMNs structures, which cannot be rapidly or fully reestablished within the predefined timeframe of our study [57]. In light of this constraint, *Glomus mosseae* was inoculated to expedite the formation of functional connectivities within the CMNs of the experimental systems. The AMF species *G. mosseae* was obtained from Nankai University (Tianjin, China) for use in the experimental trials. The inoculum was propagated for 4 months in the greenhouse using *Festuca arundinacea* and *Sorghum bicolor* as host plants. The resulting AMF inoculum consisted of a heterogeneous mixture containing viable arbuscular mycorrhizal fungal spores, extraradical mycelial networks, and colonized root fragments [53]. To determine total spore count, the inoculum was processed via a stepwise sieving protocol using a gradient of sieves with diminishing mesh sizes, enabling selective isolation of AMF spores from other inoculum constituents. The separated spores were then quantified by stereomicroscopic examination, following established protocols for mycorrhizal research to ensure data reliability [58]. For *G. mosseae*, stereomicroscopic enumeration revealed a spore density of 280 viable spores per 100 g of dry inoculum, consistent with the quantitative protocol established earlier in this study.

### 4.2. Experimental Design

Pots (18 cm height × 20 cm diameter) were used to cultivate *M. sativa*, the host plant selected for the AMF association study. A total of 2.3 kg of soil was thoroughly mixed with the GO powder to achieve final GO levels of 0% (T0, control), 0.3% (T1), and 0.6% (T2, *w*/*w*), which match the levels used in a relevant study [59]. We adopted relatively high concentrations, which might occur in accidental release or disposal sites, to elicit a dose-dependent response and better understand the mode of action. Each treatment, including the control, was replicated four times to account for inherent variability in the experimental system. To inoculate the AM fungus, 200 g of the prepared inoculum was evenly placed in the middle of the soil substrate in each pot. Surface-sterilized *M. sativa* seeds were then sown directly onto the top soil layer. The inoculated pots were subsequently transferred to a climate-controlled greenhouse facility, where environmental parameters—including temperature, relative humidity, and photoperiod—were precisely regulated to maintain optimal growth conditions for *M. sativa* and associated AMF. Daytime and nighttime temperatures were maintained at 25 °C and 14 °C, respectively, with relative humidity regulated within the range of 15–30%.

### 4.3. Soil Parameters

Rhizosphere soil pH was determined in aqueous extracts using a calibrated pHS-3C (INESA Scientific Instrument Co., LTD., Shanghai, China) pH meter. Total soil-borne organic acid content was quantified via the ferric hydroxamate spectrophotometric method [60], which involves esterification of organic acids to their fatty acid derivatives, followed by chelation with Fe^3+^ to form a red ferric hydroxamate complex with a maximum absorbance at 500 nm. Soil alkaline phosphatase and acid phosphatase activities were assayed by monitoring p-nitrophenol release from p-nitrophenyl phosphate substrate [61].

### 4.4. AMF Symbiosis Structure and Phosphorus Acquisition

Following 80 days of GO exposure, *M. sativa* seedlings were harvested, thoroughly rinsed with distilled water, gently blotted dry with filter paper, and subsequently separated into shoots and roots for subsequent physiological and biochemical analysis. Root growth parameters were evaluated by measuring primary and lateral root lengths using a calibrated ruler, along with enumeration of total lateral roots per plant. For biomass determination, shoot and root samples were oven-dried at 70 °C for 48 h to constant weight.

To assess mycorrhizal colonization, fresh roots were sectioned into 0.5–1 cm segments, rinsed thoroughly with distilled water, and cleared by incubating in 10% KOH solution at 90 °C for 30 min in a water bath. Root segments were acidified in 1% HCl for 15 min, then stained in 0.05% trypan blue solution at 60–65 °C in a water bath for 20–30 min [62]. Mycorrhizal colonization was quantified by assessing three parameters [63]: colonization frequency, colonization intensity, and vesicle abundance. For each treatment, thirty root segments were randomly selected, mounted on glass slides, and examined under a Nikon Eclipse E200 light microscope (Nikon, Tokyo, Japan).

For total P determination, 0.4 g of oven-dried plant material was digested with H_2_SO_4_-H_2_O_2_, followed by colorimetric analysis using the molybdenum–antimony method.

### 4.5. AMF Community Composition and Diversity in Rhizosphere Soil

Rhizosphere soil adhering to plant roots was collected, and three independent soil subsamples per treatment were combined to form a composite sample for subsequent analysis. Three GO treatments were established: control (T0), 0.3% (T1) and 0.6% (T2), each with three replicates.

Total soil DNA was extracted from 0.2 g of rhizosphere soil using the Fast DNA^®^ SPIN Kit for Soil (QBIOgene Inc., Carlsbad, CA, USA) following the manufacturer’s instructions. DNA purity was assessed using a spectrophotometer (Shimadzu, Kyoto, Japan). Subsequently, an 18S rRNA gene fragment was amplified via a two-round nested polymerase chain reaction (PCR) assay for AMF community analysis [64]. In the first round of amplification, the primers were AML1F (5′-ATCAACTTTCGATGGTAGGATAGA-3′) and AML2 (5′-GAACCCAAACACTTTGGTTTCCTTGGTTTCC-3′). In the second round, primers AMV4.5NF (5′-AAGCTCGTAG-TTGAATTTCG-3′) and AMDGR (5′-CCCAACTATCCCTATTAATCAT-3′) were used. The resulting PCR amplicons were purified by 2% agarose gel electrophoresis and subsequently subjected to high-throughput sequencing on an Illumina MiSeqPE300 platform (Illumina, San Diego, CA, USA) for AMF community profiling.

Raw sequence data were processed using Trimmomatic (v0.39) and FLASH (v1.2.7) software packages. Trimmomatic was used for quality filtering and FLASH was employed to merge paired-end reads, generating high-quality consensus sequences. High-quality sequences were clustered into operational taxonomic units (OTUs) at a 97% similarity threshold using UPARSE algorithm. Taxonomic assignment of representative sequences was performed by blasting against the SILVA database at 97% similarity. Fungal community α-diversity indices [65], including observed species (Sobs), richness estimator (Chao1), Shannon diversity index, and Simpson diversity index, were calculated using Mothur. β-diversity was assessed via non-metric multidimensional scaling (NMDS) based on Bray–Curtis dissimilarity matrices using the vegan package in R (v4.2.1).

### 4.6. Quantification of GigmPT and MsCS Genes

Total RNA was extracted from AMF-colonized roots using TRIzol^®^ reagent (Thermo Fisher Scientific, Waltham, MA, USA) and reverse-transcribed into complementary DNA (cDNA) for subsequent gene expression analysis. Quantitative PCR (qPCR) was performed to detect the expression of *GigmPT* and *MsCS* using a real-time fluorescence PCR System (LineGene 9600 plus; Thermo Fisher Scientific, Waltham, MA, USA). The primer pairs for *MsCS* were 5′-TATTGACGGTGATGAGGGGATTCTT-3′ and 5′-GATGTCCAAAACCAGAGAGCTTTCG-3′, and the primers for *GigmPT* were 5′-AGTATTAGGGTTTGGAGTGGTTC-3′ and 5′-GTTGCTTGTTCGATGTCTTGC-3′. The qPCR reaction mixture consisted of 10 μL of ChamQ SYBR Color qPCR Master Mix (2×), 2 μL of cDNA template, 0.4 μL of each primer (5 μM), and 7.2 μL double-distilled H_2_O, for a total volume of 20 μL. The reaction conditions were as follows: 95 °C for 5 min, 40 cycles of 95 °C for 30 s, 65 °C for 30 s, and 72 °C for 40 s. For absolute quantification of target gene copy numbers, standard curves were constructed using recombinant plasmids as reference standards [66]. The copy number of each target gene was expressed as gene copies per gram of AMF-inoculated root material, enabling reliable comparison of colonization levels across treatments.

### 4.7. Statistical Analysis

Data were expressed as mean ± standard deviation (SD). Statistical differences were analyzed using one-way analysis of variance (ANOVA) in SPSS 19.0 software. Differences between means were compared using Duncan’s multiple range test at a significance level of *p* < 0.05.

## 5. Conclusions

The pot-based inoculated system is a controlled proxy for naturally occurring CMNs rather than a full reconstruction of field-scale below-ground networks. The presence of GO induced alterations in the soil environment, characterized by increased pH, reduced organic acid content, and decreased alkaline phosphatase activity at the 0.6% GO concentration. Exposure to 0.6% GO significantly impaired AMF–host plant symbiosis by suppressing mycorrhizal colonization efficiency, inhibiting root morphological development, and reducing P acquisition capacity. Furthermore, GO downregulated the expression of *MsCS* and *GigmPT*, both essential for P acquisition and transport. In the mycorrhizal *M. sativa* system, GO disrupts plant P uptake by inhibiting mycorrhizal formation and function—a P stress that may subsequently impair the biological N fixation process. Collectively, our findings confirm that GO impairs the symbiotic relationship between N-fixing plants and AMF, leading to inhibition of P transport. This highlights the potential negative implications of GO exposure for nutrient acquisition capacity, emphasizing the vulnerability of their symbiosis-dependent nutrient uptake systems.

## Figures and Tables

**Table 1:** Effects of GO on AMF diversity indices in rhizosphere soil.

Treatments	Sobs	Chao1	Shannon	Simpson	Coverage (%)
T0	21.7 ± 1.24 ^c^	30.1 ± 3.46 ^b^	2.14 ± 0.3 ^b^	0.16 ± 0.04 ^a^	0.99 ^a^
T1	49.3 ± 1.60 ^a^	40.5 ± 2.12 ^a^	2.60 ± 0.1 ^a^	0.12 ± 0.01 ^a^	0.99 ^a^
T2	28.7 ± 3.50 ^b^	29.5 ± 1.30 ^b^	1.96 ± 0.1 ^b^	0.19 ± 0.08 ^a^	0.99 ^a^

Values are mean ± SD (*n* = 3). Different lowercase letters in the same column represent significant differences among treatments (*p* < 0.05) by the Duncan’s multiple range test. T0: Control; T1: 0.3% GO; T2: 0.6% GO.

## Data Availability

The datasets presented in this article are not readily available due to time limitations. Requests to access the datasets should be directed to the corresponding author.

## Abbreviations

The following abbreviations are used in this manuscript:

GO	Graphene oxide
CMNs	Common mycorrhizal networks
AMF	Arbuscular mycorrhizal fungi
ROS	Reactive oxygen species
SNF	Symbiotic nitrogen fixation
CS	Citrate synthase
T1	Treatment 1
T2	Treatment 2
LMWOAs	Low-molecular-weight organic acids
P	Phosphorus
N	Nitrogen
